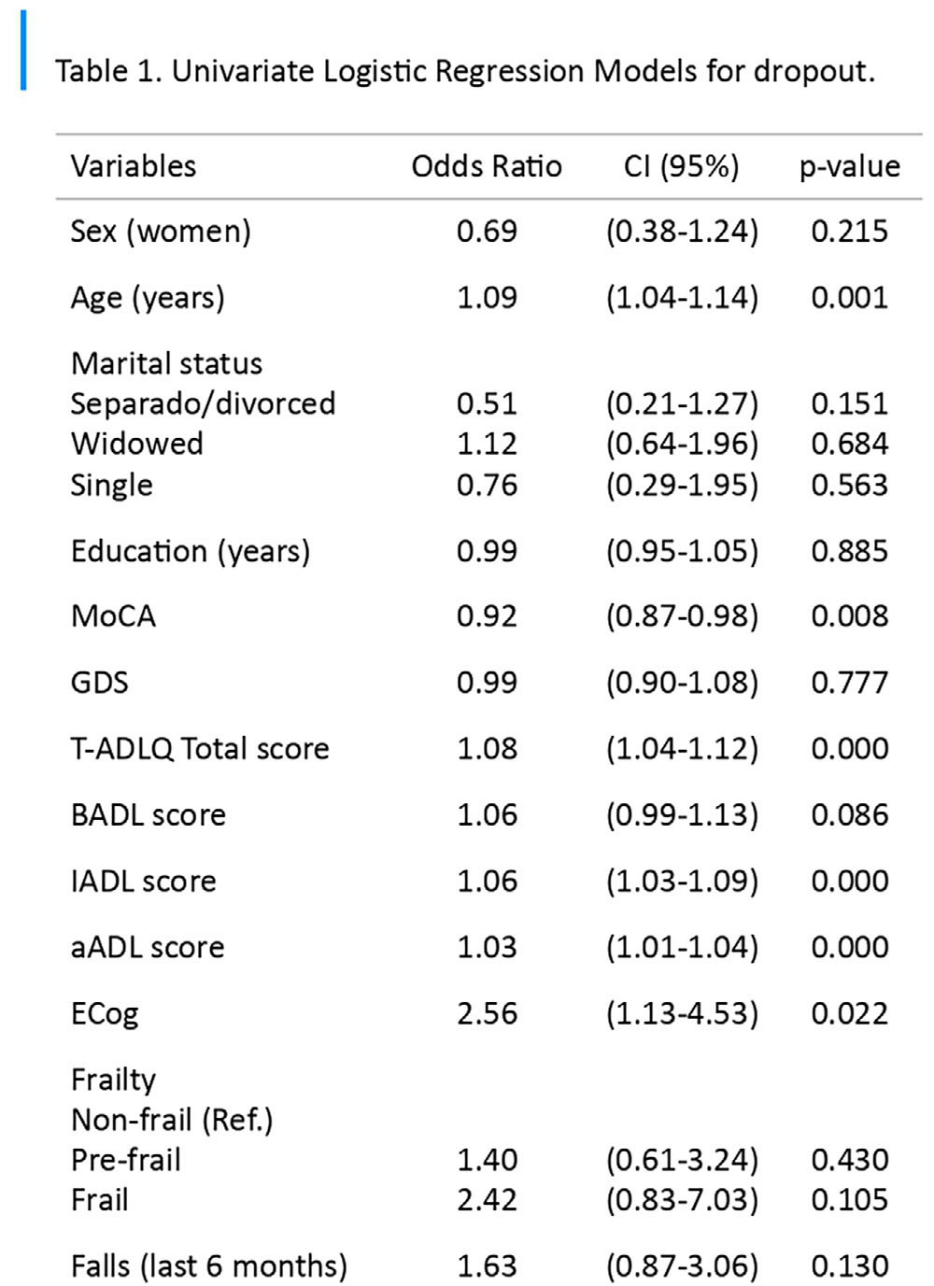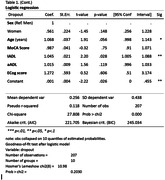# Inclusion and attrition in the community‐based cohort of functional decline of elderly with cognitive complaint of the Geroscience Center for Brain Health and Metabolism

**DOI:** 10.1002/alz.090053

**Published:** 2025-01-03

**Authors:** Teresa Parrao, Rodrigo Saguez, Daniela Thumala, Patricia Lillo, Michele Demanet, Helene Amieva, David Martínez‐Pernía, Pedro Zitko, Mauricio Cerda, Graciela Muniz‐Terrera, Andrea Slachevsky

**Affiliations:** ^1^ Universidad Alberto Hurtado, Santiago Chile; ^2^ Geroscience Center for Brain Health and Metabolism (GERO), Santiago Chile; ^3^ Department of Psychology, University of Chile, Santiago Chile; ^4^ Department of Neurology South, Faculty of Medicine, University of Chile, Santiago Chile; ^5^ Universidad de Chile, Santiago Chile; ^6^ Memory and Neuropsychiatric Center (CMYN), Neurology Department, Hospital del Salvador and Faculty of Medicine, Universidad de Chile, Santiago Chile; ^7^ University of Burdeox, Bourdeox, Bourdeox France; ^8^ Center for Social and Cognitive Neuroscience (CSCN), School of Psychology, Universidad Adolfo Ibáñez, Santiago Chile; ^9^ Biomedical Neuroscience Institute (BNI), ICBM, Faculty of Medicine, University of Chile, Santiago Chile; ^10^ CIMT Center for Medical Informatics and Telemedicine, School of Medicine, Universidad de Chile, santiago Chile; ^11^ Edinburgh Dementia Prevention, University of Edinburgh, Edinburgh United Kingdom; ^12^ Department of Social Medicine, Ohio University, Athens, OH USA; ^13^ Centre for Clinical Brain Sciences at the University of Edinburgh, Edinburgh United Kingdom; ^14^ University of Edinburgh, Edinburgh United Kingdom; ^15^ Memory and Neuropsychiatric Center (CMYN) Neurology Department, Hospital del Salvador and Faculty of Medicine, University of Chile, Santiago, Region Metropolitana Chile; ^16^ Neuropsychology and Clinical Neuroscience Laboratory (LANNEC), Physiopathology Department ‐ ICBM, Neuroscience and East Neuroscience Departments, Faculty of Medicine, University of Chile, Santiago Chile; ^17^ Neurology Department, Hospital del Salvador, University of Chile, Santiago Chile

## Abstract

**Background:**

Cognitive complaints (CC) refer to a reported experience of cognitive decline and are recognized as a potential precursor to future functional decline and progression to dementia. Identifying individuals with CC may be a valuable opportunity for preventive measures, early detection, and intervention strategies to address dementia risk. However, the characteristics of CC and its associated risk of progression to dementia are highly heterogeneous, influenced significantly by CC identification methods, recruitment approaches, and attrition in longitudinal cohort studies. This study aims to inform the inclusion and attrition processes of the GERO cohort.

**Method:**

The GERO cohort is a prospective cohort study of community‐dwelling elderly individuals with CC without dementia, aged 70 or older, living in Santiago, Chile. In this study, two samples were analyzed (dropout and non‐dropout groups) using logistic regression to assess the variables associated with attrition. Binary logistic regression analyses were conducted with dropout as the outcome variable. Prior to the multivariable models, univariate analyses were performed.

**Result:**

Out of 17,759 households approached, we successfully recruited 291 participants. Of the individuals initially recruited, 102 participants withdrew before the first follow‐up assessment. The results showed that the non‐dropout group (64.9%) was characterized by being younger (OR: 1.09; 95% CI: 1.04‐1.14), had better cognitive status according to the MoCA test (OR: 0.92; 95% CI: 0.87‐0.98), had better functionality according to the T‐ADLQ (OR: 1.08; 95% CI: 1.04‐1.12), better performance in instrumental ‐IADL‐ (OR: 1.06; 95% CI: 1.03‐1.09) and advanced ‐aADL‐ (OR: 1.03; 95% CI: 1.01‐1.04) dimensions, and better performance on the ECOG test (OR: 2.56; 95% CI: 1.13‐4.53) compared to the dropout group (35.1%). In the multivariate logistic regression model, dropout was associated with the performance of instrumental activities of daily living IADL (OR: 1.05; 95% CI: 1.01‐1.09), whereas the other variables did not exhibit statistical significance (Table 1).

**Conclusion:**

The rate of participants included, considering the number of households approached, inform on the difficulties to recruit participants with CC in the community. Functionality in daily life emerged as the key factor associated with the risk of dropout. Furthers research is needed to understand the role of functionality in CC.